# Amphibian Skin–Derived Peptides as Emerging Therapeutic Scaffolds for Metabolic Dysfunction–Associated Steatotic Liver Disease (MASLD)

**DOI:** 10.3390/ph19060962

**Published:** 2026-06-22

**Authors:** Reeju Amatya, Kyoung Ah Min, Meong Cheol Shin

**Affiliations:** 1 College of Pharmacy and Research Institute of Pharmaceutical Sciences, Gyeongsang National University, 501 Jinju Daero, Jinju 52828, Republic of Korea; reejuamatya94@gmail.com; 2 College of Pharmacy and Inje Institute of Pharmaceutical Sciences and Research, Inje University, 197 Injero, Gimhae 50834, Republic of Korea

**Keywords:** MASLD, MASH, amphibian skin secretion peptides, drug delivery, metabolic therapies

## Abstract

Metabolic dysfunction–associated steatotic liver disease (MASLD) is now the most common chronic liver disorder worldwide. Once started with hepatic steatosis, it can progress to metabolic dysfunction–associated steaohepatitis (MASH), cirrhosis, and even hepatocellular carcinoma. Insulin resistance is a major driver of hepatic lipogenesis in this disease context. Gut barrier dysfunction also contributes to the progression to MASH by allowing bacterial lipopolysaccharide (LPS) to breach into the hepatic tissues. Amphibian skin secretion peptides (ASSPs) are therefore of particular interest, given their combined metabolic and antimicrobial activities. Some ASSPs enhance glucose-stimulated insulin secretion and GLP-1 release, whereas others attenuate LPS-driven inflammatory signaling. This review introduces these ASSPs with a focus on their insulinotropic/incretinotropic and immunomodulatory activities. Also, in the latter part, pharmaceutical strategies to improve blood circulation time and structural stability would be discussed.

## 1. Introduction

**Figure 1:**
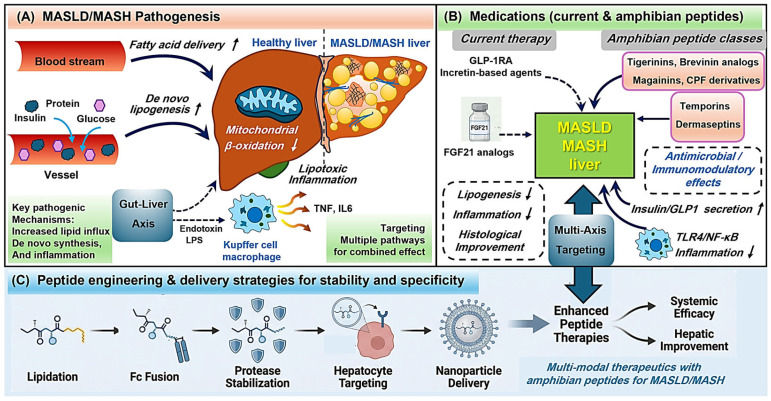
Pathogenesis of MASLD/MASH and therapeutic strategies using amphibian-derived peptides with peptide engineering approaches. (**A**) Pathogenesis of MASLD/MASH. Hepatic lipid accumulation results from increased fatty acid influx and enhanced de novo lipogenesis combined with impaired mitochondrial β-oxidation, promoting steatosis and lipotoxic stress. Translocation of lipopolysaccharide (LPS) from the gut activates hepatic immune cells via Toll-like receptor 4 (TLR4), sustaining inflammatory signaling and contributing to hepatocellular injury and progression to MASH. (**B**) Approved and investigational therapeutic strategies. Incretin-based agents-including glucagon-like peptide-1 receptor agonists (GLP-1RAs)-and fibroblast growth factor 21 (FGF21) analogs improve glycemic control, reduce hepatic lipogenesis, and attenuate inflammation. Amphibian skin secretion peptides (ASSPs) are under investigation as multifunctional candidates, exhibiting insulinotropic, incretinotropic, antimicrobial, and immunomodulatory activities. (**C**) Peptide engineering and delivery strategies. Fusion of an albumin-binding domain (ABD) or immunoglobulin Fc region, lipidation, and PEGylation extend circulating half-life, while D-amino acid substitution and peptide stapling enhance metabolic stability. These strategies aim to improve bioavailability and hepatic targeting of ASSP-based therapeutics. Upward arrow indicates increase, while downward arrow stands for decrease.

MASLD has become the most prevalent chronic liver disease worldwide. It encompasses a spectrum of pathological conditions defined by excessive intrahepatic lipid accumulation and closely linked to metabolic dysfunction [1,2]. Unfortunately, a subset of patients progresses to MASH, which can advance to cirrhosis and hepatocellular carcinoma. MASLD progression arises from multiple interacting metabolic disturbances, including increased free fatty acid influx, enhanced hepatic de novo lipogenesis (DNL), and insufficient mitochondrial β-oxidation (Figure 1) [3,4].

Insulin resistance is a central driver in this network. Hyperglycemia and compensatory hyperinsulinemia sustain lipogenic signaling, particularly through SREBP-1c, and contribute to increased triglyceride synthesis and storage in hepatocytes [5]. Additional contributions arise from the gut–liver axis. Gut-derived endotoxins and damage-associated molecular patterns (DAMP) that could reach the liver can activate Kupffer cells, promoting inflammatory cytokine production, hepatocellular injury, and hepatic stellate cell activation [6].

Therapeutically, incretin-based approaches have gained substantial attention, and indeed, there have been clinically meaningful reports. In the Phase 3 ESSENCE trial (2024/2025), semaglutide (2.4 mg) achieved MASH resolution in 62.9% of patients and was associated with significant improvements in fibrosis [7]. Similarly, the SYNERGY-NASH trial (2024) reported MASH resolution rates of up to 73.3% with the dual GIP/GLP-1 receptor agonist tirzepatide [8]. In addition, 96-week data from the HARMONY trial (2025) showed that the FGF21 analog efruxifermin can significantly reverse liver fibrosis in patients with pre-cirrhotic MASH [9]. These findings support the view that sustained correction of systemic metabolic dysfunction can translate into improvements in liver histology [10].

Despite this progress, ASSPs remain relatively underexplored as therapeutic candidates [11]. These peptides comprise a structurally diverse group of short, bioactive molecules, which were originally known for antimicrobial activity, but recently more for their insulinotropic, incretinotropic, and immunomodulatory activities [12]. Notably, these properties correspond to pathways implicated in MASLD progression.

This review will introduce the functionalities of ASSPs as enabled by their common structural features, with a specific focus on their insulinotropic, incretinotropic, and immunomodulatory activities related to the treatment of MASLD. After that, their pharmaceutical challenges will be discussed, followed by an overview of strategies to address these issues. The scheme of MASLD/MASH pathogenesis and the applicable therapeutic strategies are depicted in Figure 1.

## 2. Amphibian Skin Secretion Peptides (ASSPs): Structure and Functionality

Amphibian skin is a highly specialized secretory organ for barrier defense, osmoregulation, and chemical communication [13]. A diversity of bioactive peptides is rapidly released upon stress or injury [14], and function in the external environment, as well as permitting systemic absorption through the richly vascularized dermal network [15,16]. Structurally, most ASSPs are composed of approximately 10 to 35 amino acids. They are usually synthesized as larger prepropeptide precursors [16,17,18,19,20] and go through proteolytic cleavage or post-translational modifications [13,21,22,23]. Some of these post-translational modifications can increase the stability of the peptides. For example, disulfide bond formation, particularly within the conserved “Rana box” motif, could stabilize the C-terminal loop [13,22]. Many of these ASSPs possess amphipathic α-helical conformations [13,15], which facilitate electrostatic interaction with negatively charged microbial membranes, inducing antimicrobial activity [15,22,24].

The functionality of ASSPs is rooted in the following common structural features of short, cationic, and amphipathic sequences [13,15,16]. (1) The ASSPs possess a net positive charge, most commonly between +2 and +6 at physiological pH. Similar to cell-penetrating peptides, this cationicity appears to play a central role in initiating membrane association [25]. Positively charged residues such as lysine and arginine promote electrostatic interactions with negatively charged membrane components on mammalian cells. These interactions facilitate rapid surface binding and enable the elicitation of antimicrobial activity. Related electrostatic interactions are also implicated in their insulinotropic effects. These effects appear to arise primarily from β-cell membrane depolarization and subsequent Ca^2+^ influx [26]. In addition, several frog-derived peptides have been shown to stimulate GLP-1 secretion from enteroendocrine cells, indicating that these interactions may extend to modulation of the incretin axis [27]. However, increasing cationic charge is sometimes also related to enhanced hemolysis and nonspecific membrane disruption in mammalian cells [14,21]. As a result, if required, careful modification of surface charge remains important, particularly for applications requiring repeated or chronic administration—such as MASLD [14,21]. (2) Amphipathicity, typically expressed through α-helical organization, is another defining structural feature of the ASSPs [15,22]. Added to the cationicity that induces membrane interaction, the amphipathicity facilitates their insertion into the lipid membrane. Peptides with a well-defined hydrophobic face tend to insert more deeply into lipid bilayers, often leading to pore formation or membrane destabilization [15,22,24]. This structural feature eventually allows them to penetrate or disrupt the phospholipid bilayers, using mechanisms such as the “barrel-stave”, “toroidal pore”, or “carpet model”. (3) Another structural feature of the ASSPs is introduced through disulfide-constrained motifs, most notably the C-terminal “Rana box.” This cyclic heptapeptide element provides (1) stabilization of the alpha helical structure, (2) conformational rigidity that enhances resistance to proteolytic degradation, and (3) the source of net positive charge [20,23,28]. While the disulfide bridge is often important for antimicrobial potency, structure–activity studies indicated that its role appears context-dependent. For example, for the esculentin-1c and nigrocin-HL, truncation of the “Rana box” showed little effect on the antimicrobial activity [29,30]. These three structural determinants (cationicity, amphipathic α-helical organization, and Rana box cyclization) are introduced here as a shared framework. Their specific contributions to insulinotropic, incretinotropic, and immunomodulatory activities in individual ASSP families are discussed in Section 3 and Section 4 without further repetition of their general definitions.

## 3. Insulinotropic and Incretinotropic ASSPs

A subset of ASSPs has been shown to stimulate insulin secretion either through direct activation of pancreatic β-cells or indirectly via promotion of incretin release from enteroendocrine L cells [27,31]. Members of several peptide families, including tigerinins, brevinins, magainins, PGLAs, and CPFs, exhibit glucose-dependent insulinotropic effects in β-cell systems and improve glucose tolerance in diet-induced models of metabolic disease [26,32,33,34]. These effects are thought to involve β-cell membrane depolarization followed by Ca^2+^ influx (with or without modulation of K_ATP_ channel activity), with some peptides additionally engaging cAMP-dependent signaling pathways and enhancing endogenous GLP-1 secretion [26,27,31,34]. In GLUTag enteroendocrine L-cell models, several ASSPs, including tigerinin-1R, magainin-AM1/AM2, CPF-AM1, and PGLa-AM1, stimulate GLP-1 secretion in vitro. For example, the CPF-AM1 produced a maximal ~3.2-fold increase in GLP-1 release at 3 μM without detectable cytotoxicity in cultured L cells [27]. Comparable insulinotropic and incretin-releasing effects have been reported for tigerinin-1R and related brevinin-family analogs, which enhanced glucose-dependent insulin secretion and improved glucose tolerance in rodent models [35,36]. This is consistent with clinical data showing that GLP-1 receptor agonists can produce significant improvement in MASH. In a phase 2 trial, semaglutide achieved MASH resolution without worsening fibrosis in up to 59% of patients, compared with 17% in the placebo group [37]. Dual incretin agonism has shown similar promise, as tirzepatide produced substantial weight loss and improved biomarkers associated with steatohepatitis in clinical studies [8,38].

One mechanism through which insulinotropic ASSPs may influence MASLD is improved glycemic control and enhanced systemic insulin sensitivity. Chronic hyperglycemia drives hepatic DNL via glucose- and insulin-responsive transcriptional pathways, particularly ChREBP and SREBP-1c, leading to increased intrahepatic triglyceride accumulation [39,40]. Limiting glycemic excursions can reduce substrate availability for lipogenesis and may help alleviate lipotoxic stress. Improved insulin sensitivity may also reduce compensatory hyperinsulinemia, which otherwise sustains hepatic lipogenic signaling through the mTORC1–SREBP-1c axis and suppresses fatty acid β-oxidation via inhibition of PPARα-dependent transcription [40,41,42]. Insulinotropic ASSPs (especially brevinin- and tigerinin-derived analogs) have shown glucose-dependent stimulation of insulin secretion in vitro and improved glucose tolerance in diet-induced rodent models [13,35,36]. While direct hepatic histologic endpoints have not been assessed in these studies, improved glycemic control alone has been shown to be capable of reducing hepatic fat content in early-stage MASLD [41]. Some ASSPs also exhibit incretinotropic activity, including stimulation of GLP-1–mediated pathways [27,43]. By enhancing glucose-dependent insulin secretion, reducing postprandial glucose excursions, and slowing gastric emptying, incretin signaling helps dampen glycemic variability and downstream lipogenic drive [44,45]. Table 1 summarizes a group of ASSPs reported to exhibit insulinotropic and incretinotropic activities.

### 3.1. Tigerinins

Tigerinin-1R, first identified in the skin secretion of Hoplobatrachus rugulosus, is among the best-characterized insulinotropic peptides derived from amphibians [31,35]. In BRIN-BD11 β-cell models, it increased insulin release to ~405% of basal at 5.6 mM glucose and ~290% at 16.7 mM, without evidence of cytotoxicity under the same conditions [35]. In vivo, administration at 75 nmol/kg improved glucose tolerance and insulin secretion in high-fat diet-induced models of insulin resistance in intraperitoneal glucose challenge tests [26,35]. Arginine-substituted variant ([Arg^4^]tigerinin-1R), in particular, showed enhanced insulinotropic activity while retaining low hemolytic potential in vitro [31]. Furthermore, twice daily treatment with [Arg^4^]tigerinin-1R (75 nmol/kg body weight) for 28 days produced significantly decreased plasma glucose and glucagon concentrations, with increased plasma insulin levels [48]. Mechanistic studies indicated that tigerinin peptides acted through regulated β-cell signaling—specifically, membrane depolarization and subsequent Ca^2+^ influx [31,35,49]. In the context of MASLD, tigerinin-based peptides illustrate a viable upstream strategy: modulation of glucose homeostasis through glucose-dependent insulin secretion. Although direct measurements of hepatic triglyceride content were not reported, improved glycemic control is closely linked to reduced DNL, supporting their relevance as metabolically targeted candidates. Mechanistically, the insulinotropic action of tigerinin-1R proceeds via a K_ATP_ channel-independent pathway. Tigerinin-1R depolarizes the β-cell membrane through a mechanism that does not involve direct effects on K_ATP_ channels [35,43]. This membrane depolarization leads to the opening of voltage-dependent Ca^2+^ channels (VDCCs) and the elevation of intracellular Ca^2+^ [35]. Supporting this, electrophysiological studies with the tigerinin-1R analog [Arg^4^]tigerinin-1R demonstrated no direct effect on K_ATP_ channel activity in BRIN-BD11 clonal β-cells, whereas blockade of VDCCs or removal of extracellular Ca^2+^ markedly suppressed the insulinotropic response [48]. The response was concentration-dependent, with a significant threshold of ≥0.1 nM and a maximum of approximately 405% of the basal rate at 5.6 mM glucose [31,35]. Critically, tigerinin-1R showed no significant insulinotropic effect at the substimulatory glucose concentration of 1.4 mM, confirming strict glucose-dependency [35]. No cAMP-dependent (adenylyl cyclase–PKA) amplification has been reported for native tigerinin-1R [43].

### 3.2. Brevinins

Brevinin peptides were first identified as antimicrobial peptides in ranid frogs, including species such as Lithobates septentrionalis, but certain variants were also found to exhibit insulinotropic activity. The brevinin-2–related peptide (B2RP) increased insulin secretion in β-cell systems to ~148% of basal levels at 1 μM and 222% at 3 μM, without detectable LDH release at these concentrations [36]. In diet-induced obese mouse models, administration of B2RP analogs improved glucose tolerance and enhanced insulin responsiveness [36,50]. Structure–activity optimization has focused on reducing membrane-disruptive antimicrobial effects while maintaining insulinotropic function. Approaches such as selective truncation and targeted amino acid substitution have shown effectiveness in shifting this balance [16,50]. For example, Yao et al. designed a modified B2RP peptide with N-terminal D-amino acid substitution and C-terminal truncation, named “[D-Leu^2^]B2OS(1-22)-NH_2_”. The B2RP analog showed improved therapeutic index, which was thought of partly as the result of reduced hydrophobicity and amphipathicity by truncation of the highly hydrophobic C-terminal Rana Box region that could cause non-specific membrane disruption [51]. The insulinotropic mechanism of B2RP involves membrane-active effects. The concentration-dependent insulin release profile (148% of basal at 1 μM, rising to 222% at 3 μM in BRIN-BD11 cells), together with the absence of detectable LDH release at these concentrations, confirms that cell viability is maintained and that the response is not attributable to non-specific cytotoxic membrane disruption [36]. The concentration-response pattern is consistent with saturable membrane interaction, although no formal receptor-binding studies have been reported for B2RP. The precise signaling pathway downstream of membrane interaction, including whether K_ATP_ channel-dependent or independent components contribute, has not been explicitly characterized for native B2RP [52]. The insulinotropic activity of B2RP is therefore categorized as a direct β-cell effect, operating through Ca^2+^-dependent exocytosis.

### 3.3. Caerulein Precursor Fragments (CPFs)

The CPF peptide family, isolated from Xenopus species including X. laevis and X. amieti, has shown consistent metabolic effects in preclinical models. In diabetic (db/db) mice, [S4K]CPF-AM1 given twice daily (75 nmol/kg) over 28 days lowered fasting glucose and HbA1c, increased circulating insulin, and improved insulin sensitivity, with no detectable change in body weight or food intake [53]. The CPF-SE1 produced a similar profile in high-fat–fed mice, improving glucose tolerance, insulin sensitivity, and circulating lipid parameters [54]. Although the [S4K]CPF-AM1 study did not examine liver-specific outcomes, the reduction in HbA1c together with improved insulin sensitivity suggests a sustained shift in systemic metabolic control. In addition, CPF derivatives stimulated GLP-1 release in enteroendocrine cell models, indicating that their activity may not be limited to direct effects on β-cells but likely includes an incretin-mediated component [27]. The dual insulinotropic and incretinotropic activity of CPF peptides involves two mechanistically distinct arms. In pancreatic β-cells, [S4K]CPF-AM1 evokes membrane depolarization, increases intracellular Ca^2+^ and cAMP, and activates the protein kinase C pathway, indicating that CPF peptides engage multiple signaling pathways simultaneously in the β-cell rather than acting exclusively through a Ca^2+^-dependent mechanism [53]. This is consistent with observations on related CPF family members from Xenopus laevis, which stimulate insulin release from BRIN-BD11 cells by a mechanism that involves membrane depolarization and an increase in intracellular Ca^2+^ concentration [55]. In intestinal L cells, CPF-AM1 achieves a maximum stimulatory response of approximately 3.2-fold of the basal GLP-1 release rate at 3 μM without cytotoxicity in GLUTag cell models [27]. However, the precise intracellular mechanism by which CPF peptides trigger GLP-1 secretion from L cells has not yet been directly characterized [55]. The released GLP-1 would be expected to further amplify insulin secretion via GLP-1 receptor-coupled cAMP–PKA and Epac2 signaling in β-cells.

### 3.4. Magainins

Magainins, originally described in Xenopus laevis as amphipathic antimicrobial peptides [56], are among the best-characterized ASSPs for combined metabolic and immunomodulatory activity. On the metabolic side, magainin-AM2 stimulates glucose-dependent insulin secretion from BRIN-BD11 β-cells through a K_ATP_ channel-dependent mechanism: membrane depolarization and intracellular Ca^2+^ elevation are significantly induced, and the insulinotropic response is suppressed by extracellular Ca^2+^ chelation, diazoxide, or VDCC blockade with verapamil [34,57]. Concurrently, magainin-AM1 and -AM2, together with PGLa-AM1 and CPF-AM1, stimulate GLP-1 secretion from GLUTag enteroendocrine L cells at non-cytotoxic concentrations [27], placing magainins in the same dual β-cell/L-cell mechanistic category as CPF peptides. In high-fat–fed mice, magainin-AM2 improved glucose tolerance and insulin sensitivity compared with untreated controls [55,56]. On the immune side, magainins interact directly with LPS—molecular dynamics simulations confirm that Magainin 2 concentrates at the LPS layer of Gram-negative outer membranes, with cationic residues coordinating to phosphate groups of lipid A [58]—and experimental data show direct binding to Salmonella typhimurium LPS with consequent increase in outer membrane permeability [57]. This LPS-binding capacity, combined with GLP-1-amplifying incretinotropic activity, gives magainin-AM2 a dual metabolic–immune profile directly relevant to MASLD, in which portal LPS load activates Kupffer cells and drives hepatic inflammation while systemic insulin resistance sustains lipogenesis.

### 3.5. Peptide Glycine Leucine Amides (PGLas)

PGLa-AM1, the principal member of the Peptide Glycine Leucine Amide family from Xenopus amieti, exhibits a dual metabolic–immune profile that in several respects surpasses that of magainins. Metabolically, it stimulates glucose-dependent insulin secretion from pancreatic β-cells above 0.1 μM and GLP-1 release from intestinal L cells above 0.3 μM [43], acting via K_ATP_ channel-independent membrane depolarization with subsequent Ca^2+^ elevation—a mechanism distinct from the K_ATP_ channel-dependent pathway of magainin-AM2 and mechanistically analogous to temporins (Section 3.6). The [A14K] analog, carrying an additional positive charge at position 14, showed 2–3-fold greater potency at both β-cells and L cells and improved glucose tolerance in mice without detectable toxicity [59], demonstrating that charge-based tuning can enhance the incretinotropic component without sacrificing safety.

On the immune side, PGLa-AM1 shows the strongest LPS-binding propensity among the Xenopus-derived ASSPs tested, neutralizing endotoxin from *E. coli*, *P. aeruginosa*, and *Porphyromonas gingivalis*, and retaining broad-spectrum antimicrobial activity against oral pathogens, including Streptococcus mutans and Fusobacterium nucleatum, at concentrations non-toxic to oral fibroblasts [60]. Compared with magainin-AM2, PGLa-AM1 therefore has a better-characterized LPS-neutralizing activity and a mechanistically distinct (K_ATP_-independent) insulinotropic pathway, making it a structurally distinct but complementary candidate in the dual metabolic–immune framework relevant to MASLD.

### 3.6. Temporins

Temporins are the smallest ASSPs considered here (10–14 residues, C-terminally α-amidated) and occupy a mechanistically unique position: they are the only ASSP family that combines insulinotropic activity with immunomodulatory LPS-binding while lacking both K_ATP_ channel dependence and Ca^2+^ mobilization—properties that distinguish them sharply from magainins and PGLas (Table 1). Metabolically, temporins A, F, and G stimulate concentration-dependent insulin secretion from rat BRIN-BD11 and human 1.1B4 β-cells at concentrations as low as 1 nM without cytotoxicity up to 3 μM [47]; a broader family survey identified temporins-1Vb, -1Oe, -1DRb, and -1TGb as similarly active in the 10^−8^–10^−6^ M range [46]. Unlike magainin-AM2 (K_ATP_-dependent, Ca^2+^-mediated) and PGLa-AM1 (K_ATP_-independent but Ca^2+^-dependent), temporins produce no measurable intracellular Ca^2+^ elevation and no cAMP accumulation in β-cells, suggesting a distinct, as yet uncharacterized downstream effector pathway [46,47]. No GLP-1 secretion has been attributed to any temporin, confirming that their insulinotropic action is exclusively at the β-cell level, without the incretinotropic amplification seen in magainins and PGLas. On the immune side, temporin-1CEa suppresses TLR4/MyD88-dependent NF-κB signaling in LPS-stimulated macrophages, reducing TNF-α and IL-6 [61]. Temporin-FL neutralizes both LPS and LTA and inhibits MAPK activation in a murine sepsis model [62], and temporin-1Tl analogs show LPS-binding capacity correlating with TNF-α and NO suppression in RAW264.7 macrophages [63]. Relative to magainins and PGLas, temporins thus combine a more restricted metabolic profile (β-cell only, no incretin axis) with comparably broad immunomodulatory activity, and their small size and K_ATP_/Ca^2+^-independent mechanism make them structurally distinct engineering starting points for selective β-cell targeting in MASLD.

### 3.7. Intrahepatic Translation: From Peripheral Endocrine Effects to Hepatocyte Steatosis, Kupffer Cell Inflammation, and Stellate Cell Fibrosis

The sections above characterized ASSP effects primarily in terms of pancreatic β-cell insulin secretion and intestinal L-cell GLP-1 release. While these are the cell types for which direct in vitro and in vivo data exist, the therapeutic relevance of these peptides to MASLD ultimately depends on translating these peripheral endocrine effects into intrahepatic outcomes (specifically, reduced hepatocyte steatosis, attenuated Kupffer cell-driven inflammation, and limited hepatic stellate cell (HSC) activation and fibrosis).

At the hepatocyte level, intrahepatic fat accumulation in MASLD is driven by three converging inputs: (1) increased uptake of circulating free fatty acids (FFAs) [64,65], (2) hyperactivated DNL modulated by the transcription factors ChREBP and SREBP-1c [39,66], and (3) suppressed mitochondrial fatty acid β-oxidation [67,68]. Insulin resistance amplifies all three: it impairs suppression of adipose tissue lipolysis [64,65], potentiates ChREBP and SREBP-1c via residual insulin signaling through mTORC1 [39,66], and limits PPARα activity [67,68]. ASSP-stimulated insulin secretion and GLP-1 release address this lipotoxic issue through complementary intrahepatic actions. Improved glycemic control directly reduces ChREBP-driven DNL by lowering the hepatocyte glucose flux that activates the carbohydrate response element [39,66]. Restored insulin sensitivity reduces compensatory hyperinsulinemia and its mTORC1–SREBP-1c lipogenic drive [66]. Elevated circulating GLP-1 acts on hepatic GLP-1 receptors to activate AMPK and the downstream SIRT1 axis [69,70], suppressing SREBP-1c transcription and reducing expression of the key DNL enzymes fatty acid synthase (FAS) and stearoyl-CoA desaturase-1 (SCD1) [69,70,71], while simultaneously upregulating CPT-1α to restore mitochondrial β-oxidation [71]. GLP-1 receptor activation in hepatocytes has further been shown to reduce lipid droplet accumulation through stimulation of autophagy (lipophagy) and to alleviate ER stress, both of which reduce lipotoxic hepatocyte injury [72,73]. The most direct intrahepatic evidence for ASSPs comes from the esculentin-2CHa ABD-fusion study: in diet-induced obese mice, the long-acting SUMO-3 × ESC-ABD construct produced significant histological improvement in MASLD, with reductions in hepatic lipid content on Nile red and H&E staining, and direct inhibition of hepatocyte lipid uptake was identified as a major contributing mechanism alongside the systemic glycemic improvement. This provides proof-of-concept that an ASSP scaffold, when engineered for adequate hepatic exposure, can exert direct hepatoprotective effects at the level of the hepatocyte, not merely through secondary metabolic improvements [74].

At the Kupffer cell level, the immunomodulatory ASSPs described in Section 4.1, Section 4.2, Section 4.3, Section 4.4 and Section 4.5 act by neutralizing portal LPS before it reaches the TLR4–MD-2 complex on Kupffer cells, and by directly suppressing MyD88-dependent NF-κB and MAPK signaling in activated macrophages [75]. The intrahepatic significance of this is specific: Kupffer cells, as the liver-resident macrophages positioned in the hepatic sinusoids, are the primary first responders to portal LPS and to DAMPs released from lipotoxic hepatocytes [75,76]. Their activation drives the MASH inflammatory condition through the secretion of TNF-α, IL-1β, IL-6, and reactive oxygen species [75,76], cytokines that not only amplify hepatocyte injury but also directly suppress PPARα activity [77], thereby worsening lipid accumulation and creating a positive-feedback loop between steatosis and Kupffer cell activation. By attenuating Kupffer cell TNF-α and IL-1β output, ASSPs interrupt this loop at a hepatic-specific node. The brevinin, dermaseptin, and cathelicidin families are therefore best understood not merely as LPS-scavenging peptides, but as candidate Kupffer cell modulators whose anti-inflammatory activity has a direct intrahepatic consequence of reduced cytokine-driven hepatocyte lipotoxicity, reduced IL-1β-mediated PPARα suppression [77], and reduced NLRP3 inflammasome amplification [75].

At the hepatic stellate cell level, the fibrogenic cascade connects directly to both Kupffer cell output and hepatocyte apoptosis. Kupffer cell-derived TNF-α and IL-1β activate NF-κB in quiescent HSCs, driving expression of Col1α1 (collagen type I) and α-SMA and promoting the survival of activated myofibroblastic HSCs [78,79]. Apoptotic hepatocytes release DAMPs that are phagocytosed by HSCs, directly triggering their activation through a TLR4-dependent pathway [80,81]. LPS itself activates TLR4 on both KCs and HSCs, sensitizing the latter to TGF-β1 and inducing CCL2 secretion that amplifies the inflammatory infiltrate [82]. By attenuating LPS entry, reducing Kupffer cell cytokine output, and limiting hepatocyte apoptosis, the ASSPs reduce the three principal HSC-activating signals.

## 4. Immunometabolic ASSPs

Progression from simple steatosis to MASH reflects more than lipid accumulation alone. It involves coordinated activation of innate immune pathways, shifts in Kupffer cell phenotype, cytokine amplification, and signaling driven by gut-derived endotoxins. A subset of ASSPs, classically defined by their antimicrobial activity, is also increasingly recognized for broader immunomodulatory roles. One example is that members of the brevinin and dermaseptin families, in particular, not only elicit bactericidal effects but could also bind LPS and temper downstream inflammatory signaling. Through direct physicochemical interaction with LPS, these peptides interfere with assembly of the Toll-like receptor 4 (TLR4)–myeloid differentiation factor 2 (MD-2) signaling complex, thereby limiting downstream MyD88-dependent activation of NF-κB and mitogen-activated protein kinase (MAPK) pathways [83,84,85]. In macrophages, this is reflected by reduced phosphorylation of IRAK4, IKKβ, ERK, JNK, and p38, with consequent decreases in transcription and secretion of TNF-α, IL-6, IL-1β, and inducible nitric oxide synthase (iNOS) [85,86]. In relevant case studies, brevinin-1GHd, isolated from Hylarana guentheri, could directly neutralize LPS and suppress the release of TNF-α, NO, IL-6, and IL-1β in LPS-stimulated macrophages by inactivating the MAPK pathway [87]. Dermaseptin S4 could also bind directly to LPS and limit macrophage activation and cytokine production [88]. Similarly, temporin-1CEa and related analogs also suppress TNF-α and IL-6 secretion while downregulating NF-κB and MAPK signaling in macrophage-derived foam cells [89]. Taken together, antimicrobial ASSPs attenuate key inflammatory outputs across macrophage systems, including reduced production of TNF-α, IL-6, and IL-1β, inhibition of inducible nitric oxide synthase (iNOS), and modulation of reactive oxygen species [24,87]. These effects are consistent with pathways implicated in the progression of MASH. Peptides that neutralize LPS or dampen NF-κB and MAPK signaling may therefore help limit inflammatory escalation within the hepatic microenvironment [24,87,89]. Importantly, studies of ASSPs indicated that these ASSPs can act as immunomodulators, dampening proinflammatory signaling without causing generalized immunosuppression [84,85,86]. Beyond LPS-TLR4 signaling, the full immunopathological cascade driving MASLD-to-MASH progression encompasses hepatocyte lipotoxicity [90,91], mitochondrial oxidative stress [91,92], NLRP3 inflammasome activation [93,94], and caspase-dependent hepatocyte apoptosis [95,96], which are events that release DAMPs that activate HSCs and drive fibrogenesis through the TGF-β/Smad pathway [97,98]. While direct ASSP data on these downstream nodes remain limited, the anti-inflammatory properties of ASSPs described in Section 4.1, Section 4.2, Section 4.3, Section 4.4 and Section 4.5 are mechanistically positioned upstream of these fibrogenic signals, and several structural classes additionally show antioxidant activity [99,100] or antiapoptotic capacity [96,101] that merits consideration in this broader pathological condition. Table 2 summarizes a group of representative ASSPs with immunometabolic relevance.

### 4.1. Brevinin-1

Brevinin-1 peptides, derived from ranid frogs, are cationic amphipathic molecules that interact with both bacterial membranes and endotoxin structures. They have also been reported to bind LPS, interfere with TLR4 signaling complexes, and suppress downstream NF-κB activation [84,85,86]. Functionally, LPS neutralization translates into suppression of downstream inflammatory signaling in macrophages. In LPS-stimulated RAW 264.7 cells, brevinin-1GHd reduced the production of TNF-α, IL-6, IL-1β, and nitric oxide through inhibition of MAPK pathway activation [87]. This pathway, encompassing ERK, p38, and JNK, integrates upstream TLR4 signals and regulates transcriptional programs associated with inflammation. Similar effects have been observed with brevinin-1BW, which decreases both cytokine release and iNOS expression. The reduction in iNOS-derived nitric oxide is notable, given its contribution to oxidative and nitrosative stress in hepatocellular injury [102]. There is also evidence that some brevinin peptides act through mechanisms not limited to LPS sequestration. Studies in the Brevinin-2 subgroup indicate that certain peptides can directly modulate macrophage membrane-associated targets, leading to suppression of MAPK and NF-κB signaling independently of LPS binding [24]. Direct evaluation of brevinins in MASH histology models remains limited, but the observed suppression of TNF-α and IL-1β is mechanistically relevant. Macrophage-derived cytokines are key drivers of hepatic stellate cell activation and fibrogenesis [83,108]. By dampening this inflammatory signaling, brevinins represent a plausible approach to moderating Kupffer cell-driven amplification of inflammation during early steatohepatitis.

### 4.2. Temporins

The immunomodulatory activities of temporins, previously introduced in Section 3.6, merit emphasis in the MASLD context. Direct LPS binding, influenced by polysaccharide chain length and chemotype, interferes with TLR4 signaling at an early stage, suppressing downstream NF-κB activation and reducing systemic TNF-α and IL-6 in endotoxemia models [109]. Unlike brevinins, for which direct macrophage membrane-targeting independent of LPS binding has also been reported, the anti-inflammatory mechanism of temporins appears primarily LPS-sequestration driven. Given that portal LPS translocation is a central amplifier of Kupffer cell activation in MASH, this activity is mechanistically positioned to interrupt the endotoxin–inflammation axis even in the absence of the incretinotropic GLP-1 component present in magainins and PGLas.

### 4.3. Dermaseptins

Dermaseptins are α-helical ASSPs whose biological activity extends beyond direct antimicrobial effects to include immunomodulatory properties. Among the dermaseptin families, dermaseptin S4 is a 28-amino acid cationic ASSP (ALWKTLLKKVLKAAAKAALNAVLVGANA) originally isolated from the skin of the tree frog Phyllomedusa sauvagii. At physiological pH, it carries a net positive charge of approximately +3, conferred primarily by lysine residues, and adopts an amphipathic α-helical conformation upon association with lipid bilayers—a transition that underlies both its membrane-disrupting and immunomodulatory properties [88]. In aqueous solution, the native peptide is unstructured and prone to self-aggregation, which limits its direct antibacterial utility; optimized derivatives such as K4K20-S4 overcome this by reducing hydrophobicity, achieving MICs of 1–4 μg/mL against clinical isolates of *S. aureus* and *P. aeruginosa* and 1–16 μg/mL against *E. coli*, with substantially lower hemolytic activity than the parent compound [88]. Beyond direct microbial killing, dermaseptin S4 binds LPS and interferes with downstream inflammatory signaling—blocking LPS interaction with LPS-binding protein and suppressing cytokine production in stimulated macrophages [88]. The peptide also inhibits HIV-1 infectivity, acting directly on viral particles to disrupt virion integrity prior to host cell entry. In the context of MASLD-to-MASH progression, where portal endotoxemia and TLR4-mediated Kupffer cell activation are central to disease amplification, the capacity of dermaseptin S4 to neutralize LPS upstream of receptor engagement is of potential mechanistic relevance—though specific cytokine suppression data in hepatic macrophage models, and the concentrations needed to achieve meaningful pathway inhibition in vivo, have yet to be established.

### 4.4. Chensinin-1

Chensinin-1 is an 18–amino acid cationic antimicrobial peptide isolated from the skin secretion of the Chinese brown frog (Rana chensinensis). Its sequence (SAVGRHGRRFGL RKHRKH) carries a net charge of approximately +7 at physiological pH, a reflection of its high arginine and histidine content [110]. Biophysical studies show that it adopts a greater α-helical structure in membrane-mimetic and LPS-containing environments and binds LPS directly, with downstream consequences including reduced cytokine output from endotoxin-stimulated macrophages [110]. Structure-guided modifications have been built on this scaffold to improve potency. Substituting glycine for tryptophan and histidine for arginine in chensinin-1b (SAVWRRSRRFGLRRHRRH) raised both hydrophobicity and net positive charge, which led to tightening of endotoxin binding and more potently suppressing TLR4/NF-κB and MAPK signaling. In LPS-challenged macrophages, they could reduce TNF-α and IL-6 secretion and improve survival in endotoxemia models [110]. Chensinin-1b also shifted macrophage polarization, reducing proinflammatory markers and elevating anti-inflammatory mediators in a manner consistent with NF-κB and MAPK inhibition [110]. Regarding MASH, this class of peptides may serve as potential immunometabolic agents.

### 4.5. Cathelicidin-PP

Cathelicidin-PP is a 32-amino acid cationic ASSP (ASENGKCNLLCLVKKKLRAVGNVIKTVVGKIA) originally isolated from the skin secretion of the tree frog Polypedates puerensis [111]. At physiological pH, it carries a net charge of approximately +6 and adopts an amphipathic α-helical conformation in membrane-mimetic environments [111]. Antimicrobial potency is broad-spectrum, with minimum inhibitory concentrations in the low micromolar range (2–8 μM against Gram-negative bacteria), and hemolytic activity remains modest (below 10% at 50 μM against human erythrocytes) [111]. Beyond direct microbial killing, the peptide binds LPS and attenuates the inflammatory signaling. In murine macrophage models, Cathelicidin-PP reduced TNF-α and IL-6 secretion by approximately 40–70% at 5–10 μM and dampened TLR4–NF-κB pathway activation [111].

### 4.6. Oxidative Stress, Hepatocyte Apoptosis, and Hepatic Stellate Cell Activation: Gaps and Prospects for ASSPs

A critical gap in the current ASSP literature is the limited evaluation of these peptides against the oxidative stress and hepatocyte injury axes of MASLD pathogenesis. In the progression from steatosis to MASH, hepatocellular lipid overload impairs mitochondrial electron transport and drives the generation of ROS through NADPH oxidase (NOX) isoforms and fatty acid β-oxidation intermediates [91,112]. This oxidative burden overwhelms the endogenous antioxidant defense (principally the Keap1–Nrf2 axis, which, under physiological conditions, activates heme oxygenase-1 (HO-1), NAD(P)H quinone dehydrogenase 1 (NQO1), and glutamate-cysteine ligase) to restore redox balance [113,114]. When ROS burden exceeds the Nrf2-mediated compensatory capacity, it activates stress kinases, including JNK and p38, promotes endoplasmic reticulum stress, and initiates mitochondrial intrinsic apoptosis in hepatocytes through the Bax/Bcl-2/caspase-9/caspase-3 pathway [115,116]. In addition, the NLRP3 inflammasome is assembled and activated by ROS, oxidized lipids, and cholesterol crystals acting as DAMPs, producing IL-1β and IL-18 that further amplify hepatocyte injury [93,117]. Several ASSP classes possess physicochemical features compatible with ROS scavenging. Brevinin-1FL, isolated from the skin of *Fejervarya limnocharis*, provides a direct example. It concentration-dependently scavenges ABTS^+^, DPPH, NO, and hydroxyl radicals in cell-free assays, and in H_2_O_2_-stressed PC12 cells, it reduces MDA and intracellular ROS levels, restores superoxide dismutase and catalase activity, and attenuates caspase-dependent apoptosis [118]. In the same study, brevinin-1FL restored glutathione (GSH) content and reduced malondialdehyde (MDA) accumulation in a carrageenan-induced inflammatory model, indicating that its antioxidant activity is pharmacologically relevant in vivo [118]. Broader surveys of ranid frog skin secretions have further identified members of the temporin, brevinin-1, and brevinin-2 subfamilies with radical-scavenging capacity against ABTS and DPPH [101,119], indicating that antioxidant activity is not confined to one class but rather reflects a recurring property of the amphipathic α-helical scaffold [58]. These findings raise the hypothesis that certain ASSPs, beyond neutralizing LPS, may directly limit ROS-driven hepatocyte injury.

The downstream consequences of unresolved hepatocyte apoptosis and oxidative injury converge on HSC activation. Quiescent, lipid-storing HSCs transdifferentiate into myofibroblast-like cells upon stimulation by TGF-β_1_ (released primarily from activated Kupffer cells and injured hepatocytes), PDGF, and DAMPs derived from apoptotic hepatocyte bodies, which can be phagocytosed by HSCs to directly trigger their activation [98,120]. Activated HSCs upregulate α-SMA, produce fibrillar collagen I and III, and suppress matrix metalloproteinase activity, leading to irreversible ECM deposition if the stimulus persists [98,120]. The canonical fibrogenic signal, TGF-β_1_, operates primarily through phosphorylation of Smad2/3, formation of a Smad2/3–Smad4 complex, and nuclear translocation to drive profibrotic gene transcription [97]; ROS generated within activated HSCs via NOX4 further amplify this Smad-dependent program [121,122]. From the ASSP perspective, the LPS-neutralizing and NF-κB-suppressing activities documented for brevinins, dermaseptins, chensinin-1, and cathelicidin-PP (Section 4.1, Section 4.2, Section 4.3, Section 4.4 and Section 4.5) are mechanistically upstream of TGF-β_1_ release. By attenuating Kupffer cell-derived TNF-α and IL-1β, these peptides may indirectly reduce HSC-activating paracrine signals. Future studies should evaluate whether ASSP treatment reduces α-SMA expression, collagen deposition, and Smad2/3 phosphorylation in TGF-β_1_-stimulated HSC systems, and whether the Nrf2-activating and antiapoptotic properties observed for brevinin-class peptides translate to protection against lipotoxicity-driven hepatocyte injury in palmitate- or free cholesterol-loaded hepatocyte models. Addressing these gaps would substantially strengthen the case for ASSPs as immune-metabolic regulators capable of interrupting the full pathological chain (from portal endotoxemia and oxidative stress through hepatocyte apoptosis and stellate cell activation).

## 5. Delivery and Engineering Strategies for ASSPs

Translating ASSPs into viable therapeutics for MASLD requires overcoming a layered set of barriers that extend well beyond pharmacokinetics alone. An objective assessment identifies at least five translational gaps. First, native ASSP scaffolds undergo rapid renal clearance and proteolytic degradation, yielding plasma half-lives of minutes that are incompatible with chronic hepatic exposure [123]. Second, the cationic amphipathic architecture underlying pharmacological activity simultaneously drives concentration-dependent hemolysis, a liability unresolved for most family members at therapeutically relevant doses. Third, all in vivo ASSP data derive exclusively from small rodent models; no large-animal (e.g., non-human primate or porcine) MASLD model data exist, leaving the translational fidelity of rodent findings unconfirmed. Fourth, no ASSP study has demonstrated liver-targeted delivery. The ESC-ABD study [74] achieved hepatic benefit through systemic glycemic correction but did not employ active hepatotropic targeting or measure intrahepatic peptide concentrations. Fifth, no long-term safety data exist in any chronic dosing paradigm; the longest reported treatment window is 28 days [26,48,53], with no information on sustained immunogenicity, organ toxicity, or metabolic adaptation over clinically relevant timescales. Together, these gaps place the field at an early-to-mid preclinical stage, having established rodent proof-of-concept but lacking the large-animal, liver-targeted, and chronic safety datasets required before first-in-human evaluation. To address these obstacles, researchers have explored engineering strategies including albumin-binding domain fusion, lipidation, Fc fusion, and PEGylation to extend systemic half-life, alongside D-amino acid substitution, cyclization, and hydrocarbon stapling to enhance proteolytic resistance and conformational stability. These approaches could offer a framework for converting pharmacologically promising but short-lived peptides into therapeutic candidates for the treatment of MASLD. A pertinent real-world illustration of this translational pipeline is provided by the radiolabeled FROP peptide, where HYNIC conjugation and ^99^mTc radiolabeling transformed a short breast tumor-targeting peptide into a stable, functional radiopharmaceutical with enhanced in vivo performance and retained targeting specificity [124]. Although the application domain is oncological imaging rather than MASLD, this example directly demonstrates how targeted chemical modification and conjugation can extend effective half-life, improve tissue-targeting precision, and preserve biological activity—precisely the pharmaceutical outcomes sought for ASSP-based therapeutics.

### 5.1. Half-Life Extension Strategies

Generally, small native peptides are cleared rapidly through renal filtration, while they are also highly susceptible to proteolytic degradation during blood circulation. For a chronic condition such as MASLD, effective therapy would inevitably require sustained systemic exposure, which is difficult to achieve by unmodified peptides. This limitation is evident across various families of the ASSPs. Insulinotropic peptides, such as esculentin-2CHa derivatives, exhibit robust glucose-dependent insulin secretion in vitro and improved glucose tolerance in rodent models [125], but their short plasma half-lives severely limit their translational potential [13]. Similarly, antimicrobial peptides, including dermaseptins and temporins, are also characterized by short plasma half-lives and dose-dependent hemolytic effects [99,126]. To improve the druggability of the ASSPs, there have been many research efforts to overcome the obstacles.

Recently, Lee et al. developed a genetically engineered long-acting esculentin-2CHa(1–30) (ESC) fusion protein. Native esculentin-2CHa has insulinotropic and antihyperglycemic activity but is limited by very short systemic exposure (plasma half-life: 1.7 min) [35,123]. The architecture of the fusion protein consisted of esculentin-2CHa and an albumin binding domain (ESC-ABD), which showed markedly extended plasma persistence via FcRn-mediated recycling in murine models [74]. Based on the significantly extended circulating properties (plasma half-life: 12 h), the ESC-ABD produced sustained antihyperglycemic effects, as well as significant improvement in MASLD in diet-induced obese mice [74]. These results demonstrated how fusion-based design can convert a short-lived peptide into a longer-acting drug candidate for the treatment of MASLD.

Lipidation provides an alternative and clinically validated strategy for extending half-life. Similar to the ABD, the conjugation of long-chain fatty acids could also promote attached peptides or proteins binding to albumin, which eventually allows FcRn-mediated recycling [127]. This approach underpins the design of semaglutide, a C18-lipidated GLP-1 analog developed for once-weekly dosing [127]. Clinical studies have demonstrated prolonged exposure with acceptable tolerability [128], and in patients with biopsy-confirmed MASH, semaglutide reduced hepatic steatosis and achieved MASH resolution without worsening fibrosis in a phase 2 trial [37]. Although ASSPs differ structurally from GLP-1, this lipidation strategy can be broadly applied across different peptides to extend the plasma half-lives [127].

The Fc fusion strategy also exploits the FcRn recycling to prolong circulation time, providing a practical means of extending the half-life of therapeutic proteins [129]. When a biologically active peptide or protein is fused to the IgG Fc region, the resulting construct is subject to the pH-dependent FcRn-mediated recycling mechanism, leading to extended blood circulation time. A successful story of this Fc fusion strategy would be the case of FGF21 analog, efruxifermin. In a randomized phase 2a trial, the efruxifermin reduced liver fat content and improved the fibrosis stage of enrolling patients with MASH, offering tangible clinical evidence that half-life extension through Fc fusion can indeed produce promising therapeutic effects for the treatment of MASLD [130].

A traditional strategy for prolonging the plasma circulation of peptides/proteins has been PEGylation. The conjugation of polyethylene glycol (PEG) physically increases the hydrodynamic radius of the molecules (which prevents rapid renal clearance), while preventing opsonization during the circulation (which prevents phagocytosis by the macrophages) [131]. Although there has not been a case study for the PEGylation of ASSPs, pegozofermin has been a successful drug candidate for the treatment of MASLD. Pegozafermin is a novel, long-acting fibroblast growth factor 21 (FGF21) analog engineered with glycoPEGylation technology. In the pivotal Phase 2b ENLIVEN trial, treatment of pegozafermin 30 mg weekly and 44 mg every two weeks achieved fibrosis improvement rates of 26% and 27%, respectively, versus 7% with placebo, and MASH resolution rates of 23% and 26%, respectively, versus only 2% with placebo, with benefits sustained through 48 weeks in the blinded extension phase [132]. As the aforementioned strategies have been adopted successfully for various peptides/proteins, to date, they would be likely applicable for the ASSPs.

Beyond these established strategies, PASylation has emerged as an innovative approach for half-life extension that may complement or surpass classical methods. PASylation involves the genetic fusion of a biologically active peptide or protein with conformationally disordered amino acid sequences composed of proline (P), alanine (A), and serine (S) residues. These repetitive, hydrophilic PAS sequences adopt a random coil conformation in aqueous solution, markedly increasing the hydrodynamic volume of the fused molecule and thereby slowing renal filtration without relying on albumin recycling or chemical conjugation. Unlike PEGylation, which employs synthetic polymers that may trigger anti-PEG immune responses and can compromise biological activity through steric hindrance, PASylation is entirely genetically encoded and biodegradable, offering a fully recombinant production route with reduced immunogenicity concerns. Furthermore, PASylation has been shown to confer enhanced resistance to proteolytic degradation, which addresses one of the key liabilities of native ASSPs [133].

### 5.2. Structural Stabilization

Improving protease resistance and conformational stability is critical for the utility of ASSPs in therapeutic applications. Generally, peptides are highly susceptible to proteolytic degradation in biological environments, which significantly limits their half-life and bioavailability. One well-established strategy to address this issue is the incorporation of D-amino acids. Since most proteolytic enzymes are stereospecific and have evolved to cleave L-amino acid peptide bonds, substitution with D-isomers can substantially increase resistance to enzymatic degradation while retaining biological activity [134]. This was demonstrated by Vasu et al., who conducted an in vitro plasma degradation study examining esculentin-2CHa(1-30) and its analogs. Substitution of only three amino acids with their D-isomers (at positions 7, 15, and 23) was sufficient to markedly improve proteolytic stability. The native peptide underwent 93% degradation following 8 h of incubation with mouse plasma, while the D-amino acid-substituted analog showed only 24% degradation under identical conditions. Notably, these substitutions were spaced at intervals along the sequence rather than clustered, suggesting that positioning D-isomers at sites of preferential proteolytic cleavage may maximize stability gains without disrupting the peptide’s overall conformation or bioactivity [135].

Conformational stability can also be reinforced through complementary modification strategies, including peptide cyclization or hydrocarbon stapling. These approaches reduce structural flexibility and limit protease accessibility [99,126]. Many ASSPs naturally employ cyclization as a key mechanism for structural stability. The cyclization patterns observed range from classical disulfide bridging between cysteine pairs to more complex arrangements involving backbone circularization and side-chain crosslinking through lysine-aspartate lactam formation. Research on ASSPs from Rana species has demonstrated how disulfide constraints create stable β-hairpin motifs that maintain membrane-active conformations. The brevinins and esculentins exemplify this approach, as both of the families possess two conserved cysteine residues encompassing five amino acids forming an intramolecular disulfide bond at the C-terminus (so-called the “Rana Box”) [136]. This cyclization is considered essential to preserve the amphipathic character necessary for bacterial membrane disruption. In this regard, Chen et al. reported interesting study results. For the Brevinin-1GHa, when the Rana Box was either removed (named “Brevinin-1GHb”) or translocated to the middle of the peptide (named “Brevinin-1GHc”), antimicrobial activity dramatically changed. In the case of the Brevinin-1GHb, the spectrum of the antimicrobial activity got narrower, and for the Brevinin-1GHc, the activity declined. These results suggested the significance of the C-terminal existence of the Rana Box [137]. However, the influence of Rana Box on antimicrobial activity seemed to be different among the ASSP families. For example, according to the study by Kang et al., regarding esculentin-1c, the N-terminal residues appeared to elicit antimicrobial activity regardless of the presence of the Rana Box. Based on the study results, they concluded that the Rana Box in the esculentin-1c may contribute only to the structural stability [29].

In aqueous environments such as blood plasma, the ASSPs lose their bioactive α-helical structure, diminishing effective concentration at the target site. Hydrocarbon stapling covalently locks the helix, delivering the peptide in a pre-organized, binding-competent state. The hydrocarbon bridge also resists proteolysis on two fronts—sterically occluding protease access to the backbone and suppressing the flexible, extended conformations that serum endopeptidases preferentially cleave. The nonpolar character of the staple further raises lipophilicity and facilitates cellular uptake. The 13-residue antimicrobial peptide Temporin L has been modified using an i, i + 4 hydrocarbon staple introduced between residues 3 and 7, imposing a conformational constraint that stabilizes its α-helical structure over roughly half of the sequence [138]. This cross-link restricted backbone flexibility and fixed side-chain orientation. Within this constrained scaffold, the hydrophobic core required for membrane interaction was preserved. When tested against methicillin-resistant Staphylococcus aureus (MRSA), stapled variants showed antimicrobial activity comparable to the native peptide. The distinction was observed in biofilm models. In contrast to unmodified Temporin L, which showed limited penetration through the extracellular matrix of mature biofilms, the stapled analogs exhibited improved access to deeper layers [138]. Mourtada et al. designed various stapled antimicrobial peptides based on Magainin 2 (named “StAMPs”) [139]. Their central finding was that introducing two all-hydrocarbon staples—“double-stapling”—into optimized sequences improved multiple key properties. The lead compound, Mag(i + 4)1,15(A9K), showed superior properties to both the linear Magainin 2 and single-stapled variants. The double-stapled peptide showed near-complete resistance to broad-spectrum protease digestion and extended plasma half-life (from 2 h to over 6 h). More importantly, the Mag(i + 4)1,15(A9K) showed enhanced antimicrobial activity with less potency for hemolysis.

### 5.3. Structure-Strategy Compatibility: Matching Engineering Approaches to ASSP Classes

Lipidation is best suited to peptides that carry a free, solvent-exposed terminus or a reactive lysine side chain that is not part of the pharmacophoric helix, and whose net positive charge is moderate (+2 to +4). The mechanism of half-life extension through lipidation relies on non-covalent albumin binding driven by the fatty acid chain [140]. This interaction is charge-independent, meaning that lipidation is in principle applicable across a range of charge densities. However, for highly cationic peptides (net charge +6 to +7, such as cathelicidin-PP [net charge +6] [111] and chensinin-1b [net charge +7 at neutral pH] [141]), the attachment of a hydrophobic fatty acid chain to an already amphipathic, membrane-active scaffold can substantially amplify non-selective membrane disruption and hemolytic activity, since both the cationic face and the lipid chain independently favor erythrocyte membrane interaction [142]. By contrast, for moderate-charge insulinotropic peptides such as esculentin-2CHa derivatives and PGLa-AM1, which possess a defined hydrophilic N-terminus suitable for fatty acid conjugation without disrupting the C-terminal helix, lipidation may be a structurally sound strategy [140]. The ABD-fusion approach demonstrated with esculentin-2CHa exemplifies the same principle in a different format. Fusion at the C-terminus preserved the N-terminal insulinotropic helix and achieved plasma half-life extension to 12 h with retained antihyperglycemic activity [74].

Fc fusion imposes a substantially larger structural burden than lipidation. The IgG Fc domain adds approximately 50 kDa to the construct [143] and demands that the active peptide domain retain its conformation and receptor-binding geometry when presented as a rigid N- or C-terminal extension of a dimeric protein. This strategy is therefore most compatible with longer ASSPs (≥20 residues) that adopt stable, independently folded helical conformations in solution (such as the CPF and PGLa families, or dermaseptins [27–34 residues] [144]), where the active helix can fold autonomously without requiring the remainder of the molecule for structural support. For short peptides (≤14 residues) such as tigerinins and temporins, Fc fusion may be more problematic. The small pharmacophore may be sterically occluded by the bulky Fc domain, particularly when the bioactive residues are distributed along the full peptide length rather than concentrated at one terminus. Tigerinin-1R, whose 12-residue disulfide-constrained sequence [145] positions all pharmacophoric residues within the cyclic Rana box motif at the C-terminus, illustrates a case where N-terminal Fc attachment would be structurally preferable, but even then, the conformational context imposed by the Fc dimer interface could interfere with the membrane depolarization mechanism involving K_ATP_ channel blockade that drives its insulinotropic activity [146]. Fc fusion also carries an immunogenicity concern shared with all fusion protein approaches. The Fc region, while human-derived in clinical constructs, can still elicit anti-idiotype responses under chronic dosing conditions [147].

PEGylation and PASylation both increase the hydrodynamic radius to limit renal clearance [148,149], but they interact differently with the cationic amphipathic scaffold. PEGylation attaches a neutral, flexible polymer that sterically shields the peptide surface [148]. For highly cationic ASSPs, this shielding can partially mask the electrostatic interactions that drive membrane association, potentially attenuating both hemolytic activity and, if the conjugation site is poorly chosen, pharmacological potency [142,145]. The optimal PEGylation site for ASSPs is therefore not the charged face of the helix but a solvent-exposed position on the hydrophilic face or at a terminal region that does not participate in receptor contact. This constraint is more easily satisfied for longer peptides (temporins and PGLa analogs at ≥14 residues) with a clearly defined hydrophilic face, and more difficult for short, uniformly amphipathic peptides such as tigerinins, where nearly every residue contributes to the active conformation [145]. PASylation, being a genetic N- or C-terminal fusion rather than a site-specific chemical modification, avoids the regiochemistry problem entirely [149]. The intrinsically disordered PAS domain is appended at a defined terminus and does not interact with the amphipathic helix. This makes PASylation structurally more predictable across ASSP classes, with the principal design consideration being whether the fusion terminus is distal to the pharmacophoric segment. For Rana-box-bearing peptides (brevinins, esculentins) whose C-terminal disulfide constrains the active conformation [137], N-terminal PASylation is the geometrically preferred option. For temporins whose N-terminal residues drive membrane insertion, C-terminal PASylation should be evaluated first [99].

D-amino acid substitution and hydrocarbon stapling interact differently with the structural diversity of ASSPs. D-amino acid incorporation is charge-neutral and does not alter the net electrostatic character of the peptide, making it applicable across all charge classes without amplifying hemolytic risk [150]. The principal structural constraint is that substitutions must avoid positions critical to the active helix geometry. For peptides with a Rana box disulfide (brevinins, esculentins) [137], D-substitution outside the cyclic C-terminal motif can improve plasma stability while the constrained loop region provides independent protection against exopeptidase attack. For unstructured or partially helical peptides such as tigerinins, which rely on a specific disulfide topology for their constrained pharmacophore [145], D-substitution in the linear N-terminal segment (residues 1–10) is structurally rational and has been demonstrated to markedly reduce proteolytic degradation without loss of insulinotropic activity [146]. Hydrocarbon stapling, by contrast, requires two appropriately spaced non-natural amino acid residues (typically at i, i + 4 or i, i + 7 positions) whose side chains can bridge without disrupting the helix dipole [151]. This strategy is best suited to medium-length α-helical peptides (16–30 residues) with a continuous helical segment—conditions well met by magainins (23 residues) [56], dermaseptins (27–34 residues) [144], and the longer CPF/PGLa variants [16,152]. For short peptides such as temporins (10–14 residues), an i, i + 4 staple spans a significant fraction of the total sequence and, as the Temporin L data show, can be implemented successfully [138]. However, for near-cyclic peptides like tigerinins (12 residues with a pre-existing disulfide constraint) [145], stapling offers little additional conformational benefit over the existing cyclic scaffold and risks disrupting the pharmacophoric geometry.

## 6. Conclusions

ASSPs offer a mechanistically favorable multi-target profile for MASLD treatment, combining insulinotropic, incretinotropic, anti-inflammatory, antioxidant, and antiapoptotic activities that address the disease’s multi-hit pathogenesis. Key families stimulate GLP-1 release and glucose-dependent insulin secretion, neutralize LPS–TLR4–NF-κB signaling at the Kupffer cell interface, and may limit HSC-driven fibrogenesis via ROS scavenging and caspase suppression.

However, objective appraisal places ASSPs at an early-to-mid preclinical stage, with substantial distance from clinical application. Five barriers define this gap: (1) plasma half-lives of minutes incompatible with chronic MASLD therapy, with no engineered ASSP evaluated in large-animal pharmacokinetic models, (2) unresolved hemolytic and cytotoxic liability at therapeutically relevant doses in non-rodent species, (3) absence of liver-targeted delivery data, and intrahepatic peptide concentrations have not been measured, (4) absence of large-animal MASLD model data, as all in vivo evidence derives from murine models that imperfectly recapitulate human fibrosis progression, and (5) absence of long-term safety data beyond 28 days, with no information on anti-drug antibody formation, complement activation, or organ toxicity over clinically relevant timescales.

Bridging these gaps will require five coordinated research directions: (1) structure-guided mutagenesis to decouple insulinotropic and immunomodulatory activity from hemolytic toxicity, (2) hepatocyte-directed LNP and GalNAc/ASGPR-targeted delivery platforms with intrahepatic concentration quantification, (3) mechanistic validation in lipotoxicity-stressed hepatocytes, TGF-β_1_-stimulated LX-2 stellate cells, and Kupffer cell–hepatocyte co-culture systems, (4) family-specific pharmacokinetic engineering with formal PK–PD modeling in large-animal MASLD models, and (5) chronic safety and efficacy assessment beyond 12 weeks using MAS scoring, Sirius Red fibrosis staging, and anti-drug antibody monitoring.

## Figures and Tables

**Table 1:** Amphibian-derived skin secretion peptides (ASSPs) repurposed as metabolic and insulinotropic therapeutics.

Peptide/Family	Species Source	Length (aa)	Functional Class	Mechanistic Basis	Ref.
Tigerinin-1R	*Hoplobatrachus rugulosus*	12	Insulinotropic	Promotes β-cell membrane depolarization with subsequent Ca^2+^ influx	[35]
Brevinin-2–related peptide (B2RP)	*Lithobates septentrionalis*	21	Insulinotropic	Enhances β-cell responsiveness in a glucose-dependent manner	[36]
[S4K]CPF-AM1	*Xenopus amieti*	17	Insulinotropic /Incretinotropic	Concurrent stimulation of insulin secretion and GLP-1 release	[27]
Magainin-AM2	*Xenopus amieti*	23	Dual metabolic	Induces both insulin secretion and incretin release	[34]
PGLa-AM1	*Xenopus amieti*	22	Insulinotropic (β-cell and L-cell)	Combines membrane depolarization with cAMP-mediated signaling	[43]
Temporin variants (metabolic)	*Rana temporaria*	10–14	Emerging insulinotropic	Promotes glucose-dependent insulin secretion	[46,47]

**Table 2:** Amphibian-derived skin secretion peptides (ASSPs) relevant to hepatic innate immune modulation and MASLD/MASH therapeutic translation.

Peptide/Family	Species Source	Length (aa)	Mechanistic Features	Key Anti-Inflammatory Effects	Ref.
Brevinin-1 family	Ranid frogs	24	Interacts with bacterial endotoxin and interferes with TLR4–MD2 complex formation, limiting downstream NF-κB activation	Reduces TNF-α, IL-6, and IL-1β production	[102]
Brevinin-1GHd	Ranid frogs	24	Direct endotoxin binding suppresses inducible nitric oxide synthase and inflammatory signaling pathways	Decreases nitric oxide and proinflammatory cytokine release	[103]
Temporins	Rana temporaria	10–14	Binds LPS and dampens TLR4-mediated signaling in innate immune cells	Reduces systemic cytokine levels in endotoxemia models	[104]
Dermaseptins	Phyllomedusine frogs	27–34	Suppresses activation of NF-κB and MAPK pathways following endotoxin exposure	Limits the production of proinflammatory mediators	[105]
Chensinin-1	Rana chensinensis	18	Exhibits micromolar affinity for LPS and attenuates MAPK and NF-κB pathway activation	Improves survival and reduces inflammatory burden in LPS challenge models	[106]
Cathelicidin-PP	Polypedates puerensis	32	Modulates MAPK signaling (ERK, JNK, p38) and suppresses NF-κB activation in macrophages	Decreases nitric oxide production and inflammatory cytokine release	[107]

## Data Availability

No new data were created or analyzed in this study. Data sharing is not applicable to this article.
